# Protective Effects of Transient Glucose Exposure in Adult *C. elegans*

**DOI:** 10.3390/antiox11010160

**Published:** 2022-01-14

**Authors:** Katharina Murillo, Azat Samigullin, Per M. Humpert, Thomas Fleming, Kübra Özer, Andrea Schlotterer, Hans-Peter Hammes, Michael Morcos

**Affiliations:** 1 5th Medical Department, Medical Faculty Mannheim, Heidelberg University, 68131 Mannheim, Germany; Katharina.Murillo@medma.uni-heidelberg.de (K.M.); Kuebra.Acunman@gmail.com (K.Ö.); Andrea.Schlotterer@medma.uni-heidelberg.de (A.S.); Hans-Peter.Hammes@medma.uni-heidelberg.de (H.-P.H.); morcos@swzrp.de (M.M.); 2 Stoffwechselzentrum Rhein-Pfalz, Belchenstr. 1-5, 68163 Mannheim, Germany; humpert@swzrp.de; 3 starScience GmbH, Elisabethstr. 22, 69123 Heidelberg, Germany; 4 Department of Internal Medicine, Heidelberg University, 69120 Heidelberg, Germany; thomas.fleming@med.uni-heidelberg.de; 5 German Center for Diabetes Research (DZD), Ingolstädter Landstr. 1, 85764 Neuherberg, Germany

**Keywords:** *C. elegans*, hormesis, mitohormesis, reactive oxygen species (ROS), advanced glycation end products (AGEs), diabetes type 1, diabetes type 2, oxidative stress, hyperglycemia

## Abstract

*C. elegans* are used to study molecular pathways, linking high glucose levels (HG) to diabetic complications. Persistent exposure of *C. elegans* to a HG environment induces the mitochondrial formation of reactive oxygen species (ROS) and advanced glycation endproducts (AGEs), leading to neuronal damage and decreased lifespan. Studies suggest that transient high glucose exposure (TGE) exerts different effects than persistent exposure. Thus, the effects of TGE on ROS, AGE-formation and life span were studied in *C. elegans*. Four-day TGE (400 mM) as compared to controls (0mM) showed a persistent increase of ROS (4-days 286 ± 40 RLUs vs. control 187 ± 23 RLUs) without increased formation of AGEs. TGE increased body motility (1-day 0.14 ± 0.02; 4-days 0.15 ± 0.01; 6-days 0.16 ± 0.02 vs. control 0.10 ± 0.02 in mm/s), and bending angle (1-day 17.7 ± 1.55; 3-days 18.7 ± 1.39; 6-days 20.3 ± 0.61 vs. control 15.3 ± 1.63 in degree/s) as signs of neuronal damage. Lifespan was increased by 27% (21 ± 2.4 days) after one-day TGE, 34% (22 ± 1.2 days) after four-days TGE, and 26% (21 ± 1.4 days) after six-days TGE vs. control (16 ± 1.3 days). These experiments suggest that TGE in *C. elegans* has positive effects on life span and neuronal function, associated with mildly increased ROS-formation. From the perspective of metabolic memory, hormetic effects outweighed the detrimental effects of a HG environment.

## 1. Introduction

Diabetes mellitus (DM) (type 1 and type 2) are metabolic disorders affecting glucose metabolism, characterized by high glucose (HG) levels in the blood [1]. The major consequences of HG in humans are the development of macrovascular and microvascular complications, neuronal damage, a decreased quality of life, and finally a reduced life expectancy [2,3]. Aiming for low HbA_1c_ (glycosylated hemoglobin) targets, as a long term measure of blood glucose levels, with antihyperglycemic therapy has not consistently proven beneficial in clinical studies [4,5]. Other factors such as glucose variability [6], intraindividual differences in glucose metabolism [7], possibly as well as differences in endogenous oxidative stress compensation mechanisms [8] may play a role in the development of diabetic complications.

Some of the molecular pathways of the glucose metabolism including insulin signaling have been preserved throughout evolution and are present even in lower organisms such as nematodes or zebra fish [9,10]. *C. elegans* is an established model for studying glucose toxicity–mediated life span reduction, cellular damage and underlying molecular mechanisms [11]. It has been shown that HG conditions induce oxidative stress, which is characterized by the formation of reactive oxygen species (ROS) as well as the consecutive formation of advanced glycation end products (AGEs) leading to cellular in particular neuronal damage impacting life span [11], similar to the deleterious effects of type 1 and type 2 diabetes in humans [1]. The modifications studied in this work were associated with diabetic microvascular complications [12] in part independent from the established biomarker HbA_1c_ [13]. Fructosyllysine is an early glycation adduct and often detected in its more stable form, furosine. Methylglyoxal-hydroimidazolone and argpyrimidine are derived from the reactive glycolytic byproduct methylglyoxal and have a higher stability and half-life than fructosyllysine [14,15].

Studies examining glucose control in patients with diabetes (type 1 and type 2) suggest that some of the detrimental effects of poor glycemic control are irreversible, even when glucose is later normalized due to stricter therapy regimens [2,16]. Hyperglycemia is known to cause cellular damage, which at a certain point cannot be compensated by repair mechanisms or may even further deteriorate despite the normalization of glycemia, leading to a phenomenon called “metabolic memory”, also referred to as the “legacy effect” [17,18,19]. It is to some extent attributed to oxidative stress and AGE formation, which is accompanied by epigenetic changes and chronic inflammation [20]. In the *C. elegans* model, experiments examining a permanent HG exposure to model diabetes have consistently shown an increase in oxidative stress and AGE-formation, ultimately leading to neuronal damage and lifespan reduction [11,21,22,23,24]. On the other hand, under certain circumstances low levels of oxidative stress have been shown to elicit protective effects on an organism by triggering compensatory antioxidative mechanisms [25,26,27,28]. This effect has been described in *C. elegans* upon transient high glucose exposure (TGE) at a larval stage [29] as well as upon induction of low levels of oxidative stress, which remain under a critical temporal and quantitative threshold wherein the antioxidative compensation is exhausted and deleterious effects of ROS such as subsequent AGE formation, cellular damage, and lifespan shortening are induced [25,26,27,28]. This overcompensation with subsequent beneficial effects on the organism by antioxidative mechanisms has been termed “hormesis” or “mitohormesis”, when mitochondria are involved [27,28]. The experiments in *C. elegans* larvae suggest that the effects of TGE could differ from permanent glucose exposure considering the legacy and hormesis effects [29]. Whether the hormesis effects extend into adulthood remained unclear. Thus, the aim of this study was to examine the effects of TGE in adult *C. elegans*.

Thus, the aim of this study was to examine whether there is a persisting effect of TGE on biochemical parameters (antioxidative system, AGE), as well as the parameters of general organ function such as motility and lifespan, in adult *C. elegans*.

## 2. Materials and Methods

### 2.1. C. elegans Maintenance

*C. elegans* were cultivated on nematode growth media (NGM) with living *Escherichia coli* bacteria (OP50) as a food source as previously described [2,3]. The formation of reactive oxygen species (ROS) was studied in the transgenic CL2166 strain, which contains a transcriptional antioxidative reporter enzyme co-expressed with a green fluorescent protein upon formation with ROS [30,31]. The wild-type strain (N2 Bristol) was used for all other experiments. All nematode populations were age synchronized. Synchronicity was maintained by decreasing fertility and blocking development from eggs to larvae with 400 µM 2′-desoxy-5-fluorouridine after nematodes reached the adult stage. Adult *C. elegans* were exposed to glucose by being placed on NGM containing 400 mM glucose for a transient time period in the beginning of the experiment (TGE). The exposure lasted one, four, or six days, after which *C. elegans* were transferred to NGM without glucose. CL2166 were exposed over one, two, three, and four days, and ROS formation was assessed on day eight of the experiment.

### 2.2. Activation of the Antioxidative Response

In the transgenic strain CL2166 dvls19[(pAF15)gst-4p::gfp::nls] a transcriptional antioxidative reporter enzyme glutathione-S-transferase 4 was coexpressed with a green fluorescent protein (GFP) upon oxidative stress and quantified using a fluorescence spectrometer (Infinite M200, Tecan, Männedorf, Schweiz) with an exciting wave length of 485 nm and an emitting wave length of 538 nm. Measurements were performed after TGE over one, two, three, or four days and following non-glucose cultivation for the same period of time (respective measurements after two, four, six, or eight days of the experiment).

### 2.3. Formation of Advanced Glycation Endproducts (AGEs)

Multiple steps of physical, chemical and enzymatic treatment were necessary to isolate and unmask the AGEs argpyrimidine (AP), fructosyllysine (FL) and methylglyoxal-hydroimidazolone-1 (MG-H1) [4,5]. These were then measured using liquid chromatography–tandem mass spectrometry (LC-MS/MS) [32,33]. Briefly, protein extracts were purified by ultracentrifugation and digested by pepsin and thymol in the presence of HCl. After buffering and further degradation by pronase and aminopeptidase the unmasked AGEs were then measured using liquid chromatography–tandem mass spectrometry (LC-MS/MS) [32,33].

### 2.4. Motility

Worm motility was examined after TGE over one, four, or six days and following non-glucose cultivation until the end of the experiment on day 12. Nematodes were placed on *E. coli* coated NGM plates, movements recorded using a digital camera (Moticam 1000, Beyersdörfer GmbH, Mandelbachtal, Germany) and analyzed with specialized software (WormTracker v2.0.25, Thomas Bornhaupt, Neustadt adW, Germany). Body motility was measured in mm/s and the bending angle average in degrees/s in relation to the body center of the nematodes.

### 2.5. Life Span

*C. elegans* were examined under the microscope daily with 50 nematodes per group from the beginning of the experiment. Animals that did not move and failed to react to stimulation with a platinum wire were regarded as dead and reported accordingly. The groups were followed up until no animals remained alive.

### 2.6. Statistical Methods

The statistical analyzes were carried out using Graph-Pad Prism v7.03 (GraphPad Software, LaJolla, CA, USA). Results are reported as mean values ± standard deviations of individual groups unless stated otherwise. Independent two-tailed t-tests and one-way ANOVA were used for between group comparisons depending on the number of groups in the comparison. Comparisons between more than two groups involved Dunnett’s or Tukey’s correction for multiple testing. When comparing multiple parameters across two or more groups, two-way ANOVAs with Sidak’s post-hoc corrections were used.

## 3. Results

### 3.1. Activation of the Antioxidative Response

**Figure 1:**
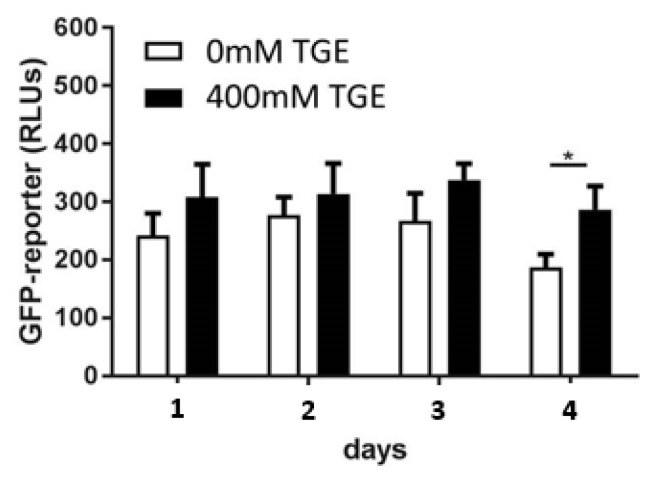
GFP-reporter for glutathione-S-transferase 4 expression after exposure to transient high glucose over indicated time-periods (day one, two, three, or four) and subsequent conduction of the experiment without TGE exposure for the same time period (measurement after a total of two, four, six, and eight days) to assess the persisting gst-4 exposure. In each experimental group the mean and standard deviation of three individual experiments is displayed with * *p* < 0.05.

In the transgenic CL2166 strain a trend of increased expression of glutathione-S-transferase 4 after all time periods of TGE could be detected measuring the GFP-coexpression (see Figure 1). A significant increase in GFP-expression was detected after the longest glucose exposure over four days following four days of non-exposure (400 mM Glc trans. 286 ± 40 RLUs vs. 0 mM Glc 187 ± 23 RLUs). These data are consistent with a threshold of glucose-mediated ROS overproduction.

### 3.2. Formation of Advanced Glycation Endproducts (AGEs)

**Figure 2:**
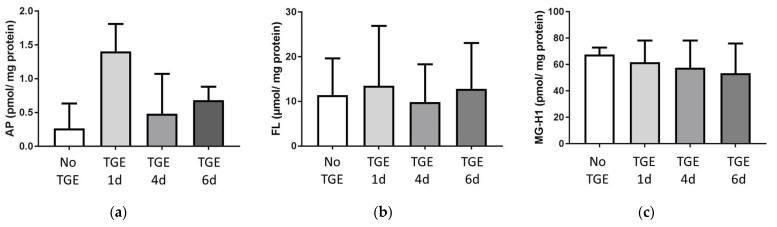
AGEs (**a**) argpyrimidine (AP), (**b**) fructosyllysine (FL), and (**c**) methylglyoxal-hydroimidazolone-1 (MG-H1), were not significantly increased on day 12 after initial TGE over 1, 4 or 6 days. In each experimental group the mean and standard deviation of three individual experiments is displayed.

In this study, TGE showed no clear effect on AGE formation after 12 days. AP formation was not affected (see Figure 2a) as well as FL and MG-H1 formation (see Figure 2b,c). This is consistent with a lack of conversion of glucose into persistent intermediate or advanced products.

### 3.3. Motility

**Figure 3:**
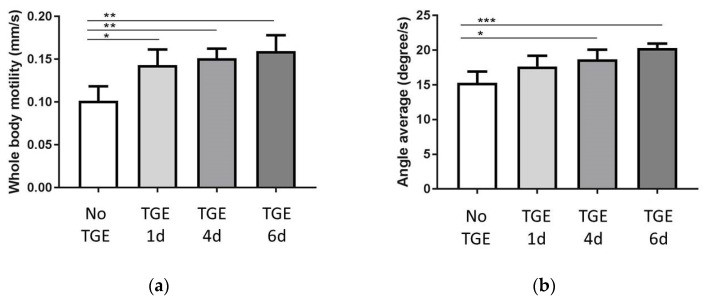
Whole body motility (**a**) and bending angle average (**b**) at the end of the experiment (12 days) after TGE over one, four, and six days. In each experimental group the mean and standard deviation of four individual experiments is displayed with * *p* < 0.05, ** *p* < 0.01, *** *p* < 0.001.

TGE led to a consistently increased motility as a marker of neuronal function of the whole body at the end of the experiment (after day 12) compared to the control group correlating with the duration of glucose exposure (1 day Glc trans. 0.14 ± 0.02 to 4 days Glc trans. 0.15 ± 0.01 to 6 days Glc trans. 0.16 ± 0.02 to 0mM Glc 0.10 ± 0.02 mm/s) The bending angle average was significantly increased upon four and six days TGE, but not upon one day TGE (1 day Glc trans. 17.7 ± 1.54 to 4 days Glc trans. 18.7 ± 1.38 to 6 days Glc trans. 20.3 ± 0.61 to 0mM Glc 15.3 ± 1.63 degree/s (see Figure 3).

### 3.4. Life Span

**Figure 4:**
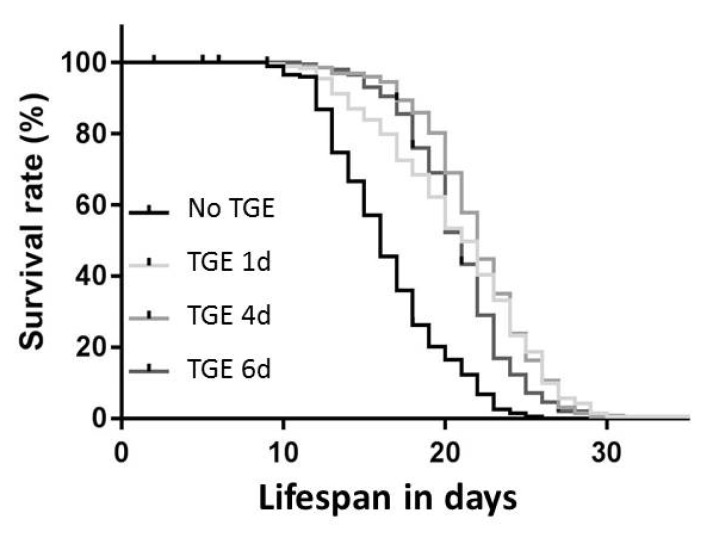
Survival rate of *C. elegans* after one, four, and six days of TGE.

*C. elegans* not exposed to HG conditions had a mean life span of 16 ± 1.3 days. TGE consistently led to a significant expansion of life span. The expansion of life span was 27% (21 ± 2.4 days) upon one-day TGE, 34% (22 ± 1.2 days) upon four-day TGE and 26% (21 ± 1.4 days) upon six-day TGE (see Table 1 and Figure 4). This set of data is unexpected and is consistent with the assumption of an (mito-)hermetic effect of TGE.

## 4. Discussion

The general effects of glucose exposure on metabolism and redox system of *C. elegans* are well described [10]. According to the data of this study, TGE appears to be beneficial to adult *C. elegans* in regards to life span and motility, which is in contrast with the previously reported effects of a persistent high glucose exposure [11,21,22,23,24]. Besides TGE, multiple supposedly deleterious exposures, which appear to increase lifespan in *C. elegans* have been described in the literature. These include, among others, the disruption of mitochondrial function, disruption of translation, disruption of insulin/ insulin-like growth factor 1 (IGF-1) signaling, caloric restriction, and exposure to xenobiotics [34]. These modulations, however, only yield life span extensions as long as their compensatory mechanisms remain intact [34]. Disrupting gene activation of protective mechanisms has been shown to invalidate the life span extension and even shorten life span in *C. elegans* (for example, for decreased insulin/IGF-1 signaling, disruption of mitochondrial function, and caloric restriction) [34].

Since the antioxidative system of *C. elegans* is likely to deteriorate with increasing age due to the general ageing processes, most severe deleterious effects of a permanent HG exposure are likely to start late in the time course of the exposure and be one factor explaining the results of this study. In our study, nematodes were exposed to HG early in life, and the exposure may not have lasted long enough to induce a decompensation of the antioxidative system (as is the case with permanent glucose stimulation).

The life span extension, with increased activation of the antioxidative system in the absence of increased AGE-levels in this study, fall in line with prior findings which have shown that a certain amount of ROS formation is necessary to extend the life span in *C. elegans* [25,35]. This also supports the idea of AGE formation as a central component of metabolic memory, which possibly initiates a self-sustaining vicious cycle, leading to inflammation and ageing [19] as it appears to be the case with permanent HG stimulation [11]. Very short exposure to TGE over one day did not result in an increase of the antioxidative response, while expanding the lifespan of *C. elegans* in this study. This indicates an earlier involvement of other antioxidative enzymes known to regulate lifespan, such as superoxiddismutases as well as possibly other cytoprotective mechanisms [30]. Further studies should investigate the role of these enzymes to dissect the complex interplay between formation of ROS, activation of the antioxidative system, and lifespan extension.

The experimental procedures in this study could also play a role explaining the results. Exposure to glucose as a solid component of NGM as opposed to application as a glucose solution on the nematodes may result in different effects, since the glucose containing NGM likely leads to less variability of the glucose concentration and thus may have limited the deleterious effects seen in prior experiments by applying a concentrated glucose solution for a short time period (11). The effect of glucose variability, which has not yet been studied in *C. elegans*, plays a significant role in the development of diabetic complications in humans and animal models triggering similar molecular pathways [6].

As a note of caution, *C. elegans* is an organism without a blood vessel system and lacking a complex organ system. Thus, extrapolation of data, obtained in nematodes such as *C. elegans*, to mammals is limited. Furthermore, this study shows first insights in the effect of TGE on adult *C. elegans,* but did not target the underlying mechanisms. For the investigation of these mechanism, further studies are needed. When exploring the effects of TGE on *C. elegans*, future research may consider evaluating the activation of transcription factors playing a central role in the *C. elegans* redox system such as SKN-1, DAF-16, and PHA-4, as well as measuring oxygen consumption and ATP-content.

## 5. Conclusions

TGE of *C. elegans* in early adult life has beneficial effects on lifespan and motility. ROS-dependent mitochondrial AGE-formation, due to permanent HG-exposure, is considered to be a primary driver behind metabolic memory and accelerated ageing. Short-time oxidative stress induction by TGE, characterized in this study by ROS formation without increased AGE-formation, is associated with observed beneficial effects. The theory of mitohormesis serves as a possible explanation for the underlying mechanisms of these effects.

## Figures and Tables

**Table 1:** Lifespan of *C. elegans* after TGE of one, four, and six days.

Glucose Exposure (mM)	Duration (Days)	Life Span (Days) Mean ± SD	Change in % as Compared to Controls	*p*-Value	*n*
0	0	16 ± 1.3	n. a.	n. a.	197
400	1	21 ± 2.4	27	<0.01	194
400	4	22 ± 1.2	34	<0.001	197
400	6	21 ± 1.4	26	<0.01	197

## Data Availability

The data presented in this study are available in article.
